# High intra-laboratory reproducibility of nanopore sequencing in bacterial species underscores advances in its accuracy

**DOI:** 10.1099/mgen.0.001372

**Published:** 2025-03-21

**Authors:** Mostafa Y. Abdel-Glil, Christian Brandt, Mathias W. Pletz, Heinrich Neubauer, Lisa D. Sprague

**Affiliations:** 1 Institute of Bacterial Infections and Zoonoses, Friedrich-Loeffler-Institut, Naumburger Str. 96A, 07743 Jena, Germany; 2 Institute for Infectious Diseases and Infection Control, Jena University Hospital – Friedrich Schiller University, Jena, Germany; 3 InfectoGnostics Research Campus Jena, Center for Applied Research, 07743 Jena, Germany

**Keywords:** assembly, clustering, genome, long read, nanopore sequencing, reproducibility, accuracy

## Abstract

Nanopore sequencing is a third-generation technology known for its portability, real-time analysis and ability to generate long reads. It has great potential for use in clinical diagnostics, but thorough validation is required to address accuracy concerns and ensure reliable and reproducible results. In this study, we automated an open-source workflow (freely available at https://gitlab.com/FLI_Bioinfo/nanobacta) for the assembly of Oxford Nanopore sequencing data and used it to investigate the reproducibility of assembly results under consistent conditions. We used a benchmark dataset of five bacterial reference strains and generated eight technical sequencing replicates of the same DNA using the Ligation and Rapid Barcoding kits together with the Flongle and MinION flow cells. We assessed reproducibility by measuring discrepancies such as substitution and insertion/deletion errors, analysing plasmid recovery results and examining genetic markers and clustering information. We compared the results of genome assemblies with and without short-read polishing. Our results show an average reproducibility accuracy of 99.999955% for nanopore-only assemblies and 99.999996% when the short reads were used for polishing. The genomic analysis results were highly reproducible for the nanopore-only assemblies without short read in the following areas: identification of genetic markers for antimicrobial resistance and virulence, classical MLST, taxonomic classification, genome completeness and contamination analysis. Interestingly, the clustering information results from the core genome SNP and core genome MLST analyses were also highly reproducible for the nanopore-only assemblies, with pairwise differences of up to two allele differences in core genome MLST and two SNPs in core genome SNP across replicates. After polishing the assemblies with short reads, the pairwise differences for cgMLST were 0 and for cgSNP were 0–1 SNP across replicates. These results highlight the advances in sequencing accuracy of nanopore data without the use of short reads.

**Impact Statement**

Nanopore sequencing shows great promise for clinical applications but requires extensive validation to ensure consistent and reliable results. This validation can be achieved through comparative studies with established methods, proficiency testing and both inter- and intra-laboratory comparisons. The aim of this study was to evaluate the reproducibility of nanopore sequencing under uniform conditions using a benchmark dataset of five bacterial species. We assessed reproducibility by measuring discrepancies between sequencing replicates, analysing plasmid recovery results and examining genetic markers and clustering information. Our results show high reproducibility of nanopore sequencing results in various genomic analyses, highlighting improvements in the accuracy of nanopore sequencing.

## Data Summary

The authors confirm that all supporting data, code and protocols have been provided within the article or through supplementary data files.

The sequencing data are available with NCBI BioProject accession PRJNA1206708.

## Introduction

Oxford Nanopore sequencing is a DNA sequencing technology that uses nanopores, tiny protein channels embedded in a membrane [1,2]. Changes in the electrical current occurring when a DNA molecule passes through the pore are measured and translated to identify the individual molecule. This sequencing method, in contrast to short-read methods such as Illumina, offers real-time data, portability, cost-effectiveness and long-read sequences [1,2], which enable the resolution of complex genomic regions, structural variations and repetitive sequences as well as recovery of chromosome and plasmid sequences in bacteria [3].

In outbreak and surveillance studies, the accurate identification of genetic variants is crucial for the determination of strain relatedness and necessitates artefact-free sequencing data [4]. Nanopore sequencing has faced high error rates in the past, which were as high as 10% [5]. However, ongoing improvements in nanopore sequencing – such as R10 technology, which involves an extended barrel pore design and dual reader head – and advances in software algorithms, such as duplex basecalling, where the complement strand is read immediately after the template strand, have significantly improved accuracy [3,6]. Despite these advances, certain areas remain challenging, particularly homopolymers and methylation regions [7,8].

Thorough validation of nanopore sequencing for clinical diagnostics is essential to ensure its reliability. This can be achieved through several approaches, including comparative studies where nanopore sequencing results are measured against established diagnostic methods such as PCR, Sanger sequencing or other next-generation sequencing platforms [9]. Additionally, sequencing known reference samples with well-characterized genetic variants, conducting proficiency testing and performing intra- and inter-laboratory comparisons are crucial for establishing consistency and reproducibility [10,11].

In this work, we aimed to evaluate the reproducibility of nanopore sequencing under consistent conditions, i.e. with the same chemistry, instrumentation, personnel, infrastructure and analysis workflow. We used a benchmark dataset of five bacterial species and generated eight technical sequencing replicates of the same DNA for each species. We assessed reproducibility by measuring discrepancies (substitution and insertion–deletion errors) between replicates, analysing plasmid recovery outcomes and examining genetic markers and clustering information across replicates. We compared the results of nanopore-only genome assemblies with and without short-read polishing. Finally, we automated our assembly workflow using a Snakemake-based approach [12].

## Methods

**Fig. 1.**
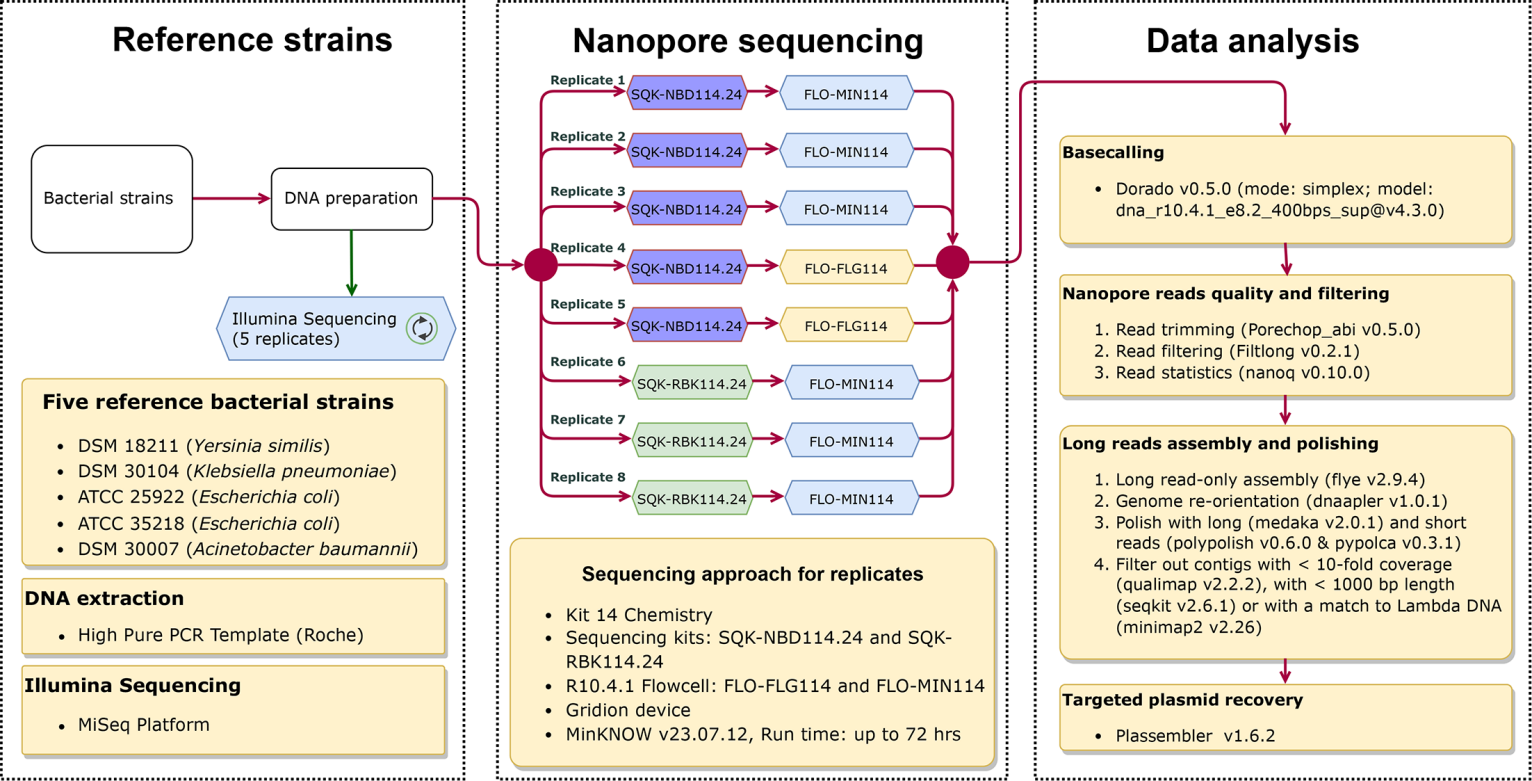
Methodological workflow used in this study.

The methodological workflow implemented in this study is shown in Fig. 1.

### Bacterial strains and DNA extraction

A panel of five reference bacterial strains was purchased from the DSMZ-German Collection of Microorganisms and Cell Cultures. The strains include one *Klebsiella pneumoniae* strain DSM 30104 (accession: GCF_006364295.1), one *Yersinia similis* strain DSM 18211 (GCF_000582515.1), two *Escherichia coli* strains, ATCC 25922 (DSM 1103, GCF_028596225.1) and ATCC 35218 (DSM 5923, no NCBI accession), and one *Acinetobacter baumannii* strain DSM 30007 (GCF_019331655.1) (Table S1, available in the online Supplementary Material).

The High Pure PCR Template kit (Roche) was used for DNA extraction from each strain according to the manufacturer’s protocol. The concentration and purity (absorbance ratios 260 out of 230 and 260 out of 280) of extracted DNA were measured using a Colibri spectrometer (Thermo Fisher Scientific, USA). The integrity of the DNA was analysed by agarose gel electrophoresis. The DNA concentration of double-stranded DNA was calculated using a Qubit 3.0 fluorometer (Life Technologies, Germany) with the Qubit dsDNA BR Assay Kit, which quantifies DNA based on the fluorescence of a dye that binds to double-stranded DNA.

### Next-generation sequencing

DNA from one preparation from each reference strain was subjected to next-generation sequencing using both Illumina (five replicates) and nanopore sequencing (eight replicates).

The eight technical nanopore sequencing replicates were performed using the R10.4.1 MinION flow cells (FLO-MIN114) for six replicates and with the R10.4.1 Flongle flow cells (FLO-FLG114) for two replicates (Fig. 1). Ligation sequencing - Native Barcoding Kit 24 (SQK-NBD114.24) and Rapid Barcoding kit (SQK-RBK114.24) were used for sequencing, following the protocol versions NBE_9169_v114_revQ_15Sep2022 and RBK_9176_v114_revO_20Nov2024, respectively (Fig. 1). Nanopore sequencing was then conducted on a GridION X5 Mk1 device using MinKNOW v23.07.12 (https://community.nanoporetech.com/) with a default translocation speed of 400 bp/s and a data sampling frequency of 5 kHz. The sequencing run times were set to up to 72 h for the FLO-MIN114 flow cell or until no more data were generated for the FLO-FLG114 flow cell. For the FLO-MIN114 flow cell, each DNA from the five strains was multiplexed and sequenced together, while for each Flongle cell (FLO-FLG114), the DNA from each sample was loaded separately. In sum, 16 nanopore sequencing libraries were prepared to generate eight technical replicates for each sample: six multiplexed libraries loaded on MinION and ten singleplex libraries loaded on Flongle (Table S2).

The Illumina paired-end sequencing library was prepared with the Nextera XT DNA Library Preparation Kit (Illumina Inc., USA) and loaded onto an Illumina MiSeq machine (Illumina Inc., USA), which generated reads of 300 bp in length (read count range 619 215–807 386) for five replicates.

### Nanopore sequence basecalling

The nanopore raw POD5 data were basecalled using the super-accuracy model (sup) with dorado v0.5.0 (https://github.com/nanoporetech/dorado). This model (sup) has been shown to provide accurate results compared to the other models (hac and fast) [13,14]. Basecalling was conducted in a simplex mode as the demultiplexing feature has not been implemented in dorado duplex as of version 0.5.0. The following command was used: dorado basecaller --kit-name <kit-name> --sample-sheet <sample_sheet> -r dna_r10.4.1_e8.2_400bps_sup@v4.3.0 <sequencing_run> > calls.bam.

For the FLO-MIN114, the dorado demux command was used for barcode demultiplexing with the flag --emit-fastq.

For the FLO-FLG114, all generated sequencing reads were concatenated per each sequencing run using the samtools v1.18 [15], and the redundant sequences were discarded with SeqKit v2.6.1 [16], with the following command: samtools fastq calls.bam | seqkit rmdup -o <sample_fastq>.

The summary files of the sequencing runs were compared with NanoComp v1.24.0 [17]. The basecalled sequencing data are available under NCBI BioProject accession PRJNA1160475.

### Development of nanobacta for automated genome assembly of nanopore data

To ensure high reproducibility in downstream analyses, we searched recent literature and GitHub repositories for best practices of the assembly of Q20+ nanopore data. Despite the availability of numerous pipelines, no well-established consensus could be found [14,18,21]. As such, we built and automated a workflow to provide a means for others to replicate our approach. We prioritized automation over manual review and individual assessment. Therefore, we did not involve software that requires manual inspection. We also used the framework of the Snakemake workflow system [12] to automate the workflow. This framework also enables the automatic deployment of software dependencies. The source code of this workflow is publicly available at https://gitlab.com/FLI_Bioinfo/nanobacta. The main analysis steps are summarized in Fig. 1 and are briefly explained below. Fig. S1 shows the directed acyclic graph of the workflow steps.

First, the nanopore sequencing reads were trimmed with porechop_ABI v0.5.0 [22] and filtered with Filtlong v0.2.1 [23] to obtain 95% of the best reads with a minimum length of 1 kb and a minimum average quality of 10. Additionally, Filtlong v0.2.1 [23] was used to down-sample the nanopore data to a maximum of 0.4 Gb per sample to achieve a theoretical coverage of 80–100-fold per sample, considering genome sizes of 3.4–5 Mb. This step of down-sampling was necessary to prevent the Flye assembly process from breaking down due to excessive coverage (>500-fold) of samples (data not shown). The sequencing statistics of the raw and filtered reads were gathered with nanoq v0.10.0 [24].

Second, long-read assembly and polishing were performed as follows: The *de novo* genome assembly of long reads was performed with Flye v2.9.4 [25]. We employed three rounds of polishing with Flye (flag -i 3) because we noticed that these additional rounds of Flye polishing helped to correct small structural errors in the ATCC 35218 sample that the subsequent short- and long-read polishers could not correct. The circularized contigs from Flye were adjusted with dnaapler v0.4.0 [26] to ensure that they start with *dnaA* for chromosomes and *repA* for plasmids. Polishing with long reads was followed with medaka v2.0.1, model r1041_e82_400bps_bacterial_methylation. We excluded Racon from the polishing process as we observed that its use resulted in an increased number of mismatches and indels in the genome (data not shown). This observation is consistent with recent guidelines recommending its omission. Although the use of medaka for polishing is controversial, we found that it provided slightly better polishing performance for nanopore data (data not shown). Therefore, medaka was employed as the sole long-read polisher in this study.

Optionally, subsequent polishing with the short reads was followed. For this, fastp v0.20.1 was used for quality filtering of the reads. Polishing was performed with two rounds of Polypolish v0.6.0 (flag --careful) [21,27] and one round with Pypolca v0.3.1 (flag --careful) [21]. The resulting polished assembly was filtered from contigs with a coverage depth <10-fold (using minimap2 v2.26 [28] for mapping and Qualimap v2.2.2 [29] to obtain mapping statistics), contigs with a length <1000 bp (using SeqKit v2.6.1 [16]) or with a match to lambda DNA, the control DNA used during sequencing with kit 14 (using minimap2 v2.26 [28] for contig alignment). The genome statistics of the final assembled data were summarized using SeqKit v2.6.1 [24]. Finally, Qualimap v2.2.2 [29] was used to estimate the average assembly read depth based on the filtered sequencing reads.

In a final step, we used Plassembler v1.6.2 [30] for targeted plasmid recovery. We used the unfiltered long reads as input for Plassembler when running in long-read-only mode (command plassembler long), as we found that Filtlong does occasionally filter out plasmid reads.

One goal of this study was to compare the combined short- and long-read approach with the long-read-only-based approach. To simplify the comparison, the short- and long-read approach was based entirely on the analysis steps described above. The long-read-only approach was also based on the analysis steps above, with the exception of the steps for short-read polishing with Polypolish and Pypolca (Fig. S1).

### Genome sequence comparison

We compared the assembly results produced from all replicates of each sample with medaka v2.0.1 (https://github.com/nanoporetech/medaka). Briefly, we used medaka tools consensus2vcf to report nt substitution and insertion or deletion (indel) variants between pairs of genome replicates. For that, the pairwise alignment was performed with the edlib library [31] using the alignment mode NW for a global alignment in both consensus and reference. Then, the command, medaka tools classify_variants, was used to classify the identified sequence variants. We also used the script error_positions.py [18] to quantify the ratio of concordance between the replicates in the repeat and non-repeat sequences [18].

### Genome characterization

We investigated the genetic features of genome assemblies as follows: We used Kraken2 v2.1.2 [32] for taxonomic classification of the genomes; checkM v1.2.2 [33] to evaluate genome completeness, contamination and heterogeneity; FastANI v1.33 [34] to estimate the average nucleotide identity (ANI) percentage relative to reference genomes; and ReferenceSeeker v1.8.0 [35] to report Mash distance, ANI and conserved DNA in relation to closely related references in the NCBI database. Additionally, we employed typing using the traditional MLST scheme using the MLST v2.22.1 tool (https://github.com/tseemann/mlst) and the PubMLST database [36].

To search for genetic markers linked to resistance, we first utilized ABRicate v1.0.1 (https://github.com/tseemann/abricate) along with internal databases, CARD [37], ResFinder [38], NCBI [39], ARG-ANNOT [40] and MEGARes [41]. All databases were last updated on 20 December 2024. ABRicate uses blastn v2.12.0+ [42] for searching the genome sequence for resistance genes without prior gene prediction. It therefore ought to be less sensitive to indel errors. Next, we searched for resistance genetic determinants using AMRFinderPlus v3.12.8 [39], database version 2024-07-22.1, with the flag –-organism activated for Escherichia, Acinetobacter_baumannii and Klebsiella_pneumoniae. The AMRFinderPlus was run with the protein and gff3 annotation files (flags, -p and -g). Finally, we used the Resistance Gene Identifier (RGI v6.0.3) from the CARD database 3.2.9 [37]. The RGI_CARD was used with Pyrodigal [43] for gene prediction.

To search for virulence-related markers, ABRicate v1.0.1 was used in combination with the Virulence Factor Database (VFDB) (last updated on 20 December 2024) [44]. Further, ECTyper v1.0.0 (database version 1.0) [45] was used for serotype prediction of the two *E. coli* strains, and Kleborate v2.3.2 [46] and Kaptive v2.0.6 [47] to characterize the *K. pneumoniae* strain with respect to virulence and resistance genetic determinants as well as the capsule and outer lipopolysaccharide loci.

We annotated all genomes with Bakta v1.5.1 [48] and used Panaroo v1.3.0 [49] with default settings to count core and total genes in default mode.

### Genome clustering

Hierarchical clustering was performed using the hclust function in R v3.6.3 [50] and the single linkage method, which is similar to the minimal spanning tree following the ‘friends of friends’ clustering strategy. A clustering threshold of five different variants was used for all datasets. Clustering was based on the pairwise differences between each genome pair calculated from either the core genome SNPs (cgSNPs) or the core genome MLST (cgMLST) data.

To determine the pairwise differences from cgSNP analysis, we first used snippy v4.6.0 (https://github.com/tseemann/snippy) to identify SNP variants in the genome. Given that the choice of reference genome can influence the results of this analysis [51,53], we used the same strain genome listed in Table S1 as the reference. For *E. coli* ATCC 35218, for which there was no freely available reference genome, GCF_001683435.1 was used as the reference based on the Mash distance estimation, indicating that this is the closest genome in the NCBI. Snippy-core v4.6.0 was then used to construct an alignment of the cgSNPs removing invariant sites and sites with indel mutations represented by gaps in the concatenated alignment. Phage and repetitive regions were not removed from the alignment. The resulting alignment file was then used with snp-dists v0.6.3 (https://github.com/tseemann/snp-dists) to estimate SNP distances between genome pairs.

To determine the pairwise differences for cgMLST, analysis was performed using chewBBACA v3 [54]. To do so, we imported the cgMLST schemes for *E. coli* [55,57], *K. pneumoniae* [57,58] and *A. baumannii* [59] from the Ridom cgMLST.org (https://cgmlst.org/ncs; accessed in August 2024) and for *Yersinia pseudotuberculosis* [60] (applied to *Y. similis*) from the Pasteur MLST website (https://bigsdb.pasteur.fr/yersinia; accessed in November 2023). All schemes were adapted with chewBBACA v3. To create training files for chewBBACA v3, Prodigal v2.6.3 [61] or Pyrodigal v3.3.0 [43] were used with the genomes GCF_008632635.1 for *A. baumannii*, GCF_000240185.1 for *K. pneumoniae*, GCF_000008865.2 for *E. coli* and GCF_000834295.1 for *Y. pseudotuberculosis*. chewBBACA AlleleCall v3.3.1 [54] was then used for allele calling, and the core genome sequence type profile for each sample was reported with chewBBACA ExtractCgMLST v3.3.1 [54]. The pairwise cgMLST allele distances were calculated using Grapetree v2.1, with missing values (uncalled alleles) being ignored in pairwise comparison [62]. This was used to create pairwise differences from the cgMLST results, which were passed to hclust (R v3.6.3) [50] to create hierarchical clusters based on these distances.

## Results and discussions

### Sequencing data characteristics

Eight replicates were produced for each reference strain: three with the Ligation Barcoding kit loaded on the FLO-MIN114 flow cell (multiplexing the five samples); two with the Ligation Barcoding kit, loaded on the Flongle FLO-FLG114 flow cells; and three with the Rapid Barcoding kit loaded on the FLO-MIN114 flow cell (multiplexing the five samples).

For the FLO-MIN114-based runs, the Ligation Barcoding kit yielded higher sequencing data throughput compared to the Rapid Barcoding kit. The Ligation kit yielded a total of 16, 20 and 27 Gb of sequence data, respectively, while the sequence output from the Rapid Barcoding kit was 8.4, 12.4 and 11.14 Gb of sequence data, respectively. In contrast, the Rapid kit yielded longer reads than the Ligation kit. The mean read length per sample with the Rapid kit was 5.5 kb, and the mean N50 read length was 13.15 kb, while the mean length per sample with the Ligation kit was 2.9 kb, and the mean N50 read length was 8.4 kb.

The unclassified reads ranged from 0.83 to 1.9 Gb, accounting for 4.9%–12.4% of all basecalled sequences. For the classified reads (i.e. identified barcoded samples), the distribution of per-barcode sequence data varied between the Rapid and the Ligation kits. With the Ligation kit, an average of 37.34% of the sequences were from the sample *Y. similis* DSM 18211 (sd 3.54), 10.45% from *K. pneumoniae* DSM 30104 (sd=1.22), 11.24% from *E. coli* ATCC 25922 (sd=0.49), 23.39% from *E. coli* ATCC 35218 (sd=0.91) and 11.49% from *A. baumannii* DSM 30007 (sd=1.13) (Tables S2 and S3). With the Rapid kit, an average of 7.52% were from *Y. similis* DSM 18211 (sd=2.24), 42.62% from *K. pneumoniae* DSM 30104 (sd=6.6), 12.67% from *E. coli* ATCC 25922 (sd=3.06), 4.6% from *E. coli* ATCC 35218 (sd=1.0) and 20.44% from *A. baumannii* DSM 30007 (sd=3.25) (Tables S2 and S3). This variation in the distribution of per-barcode sequence data was consistent across the multiplexed runs and is likely due to the DNA used and library preparation type. However, no obvious variation in the quality of the DNA samples was observed based on the DNA quality metrics implemented here.

The mean theoretical read depths per sample were 609× (range 61× to 1812×). Specifically, *E. coli* ATCC 25922 had a mean coverage of 369×, *E. coli* ATCC 35218 had a mean coverage of 541×, *Y. similis* DSM 18211 had a mean coverage of 888×, * A. baumannii* DSM 30007 had a mean coverage of 601× and *K. pneumoniae* DSM 30104 had a mean coverage of 648×.

The ten libraries sequenced on the Flongle FLO-FLG114 flow cells produced, on average, 0.72 Gb per run and sample (range 0.27–1.2 Gb) (Tables S2 and S3). The mean read length per sample was 3.1 kb, and the mean N50 read length was 9.8 kb. The mean theoretical read depth per sample was 145× (range 50× to 245×). *E. coli* ATCC 25922 had depths of 165.1× and 129.3×, and *E. coli* ATCC 35218 had depths of 173.5× and 243.6×. *Y. similis* DSM 18211 had depths of 180.6× and 103×. *A. baumannii* DSM 30007 had depths of 96.7× and 115×, and *K. pneumoniae* DSM 30104 had depths of 192.6× and 50.7×.

In all cases, the sequencing runs achieved at least a 50-fold sequencing depth per sample across the reference strain. The sequencing data from the MinION flow cells show a markedly higher throughput compared to data from the Flongle flow cells. These differences in data throughput are entirely expected given the small size of the Flongle flow cell and the ultimately small number of nanopores available for sequencing, which are suitable for sequencing only one bacterial strain, as previously described [63]. The data from both platforms, MinION and Flongle flow cells, were sufficient to generate circularized genomes as described below.

### Impact of short-read polishing on assembly reproducibility and accuracy

Nanopore sequencing has faced challenges with high error rates in the past. Since we sequenced identical DNA samples, we assumed that the obtained sequences should be identical. To assess this, we quantified discrepancies between replicates, focusing on substitution and indels. These are likely errors introduced during sequencing or sequence processing. We compared the reproducibility of assemblies polished with long reads only (i.e. without short-read polishing) to those polished with both long and short reads.

**Fig. 2.**
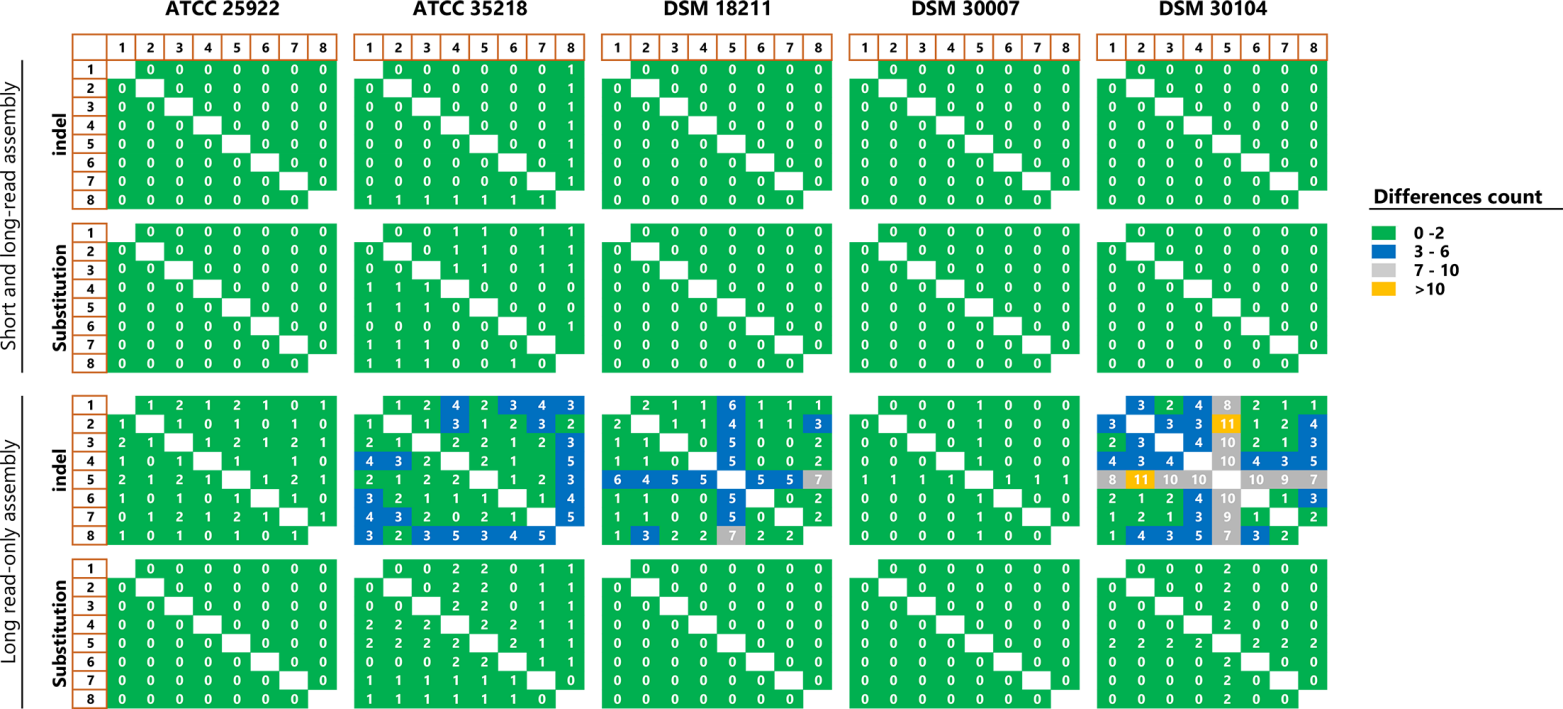
Comparison of nt substitution and insertion/deletion errors for sequencing replicates (pairwise comparison) of the five reference strains. The assembly results are compared between the long-read-only approach (without short-read polishing) and the combined short- and long-read approach.

Fig. 2 shows the total number of nt substitutions and indels in the chromosome of each pair of sequencing replicates. Assemblies polished with both short and long reads exhibited a mean nt substitution of 0.11 (sd=0.31, median=0 and max=1 substitution). Assemblies polished with long reads only showed a mean value for nt substitution of 0.31 (sd=0.67, median=0 and maximum=2). The mean difference between the two approaches was 2.0, with an se of 0.04. The 95% CI ranged from 0.11 to 0.28, with a t-test confirming the significance of this difference (*P*<0.0001).

With respect to gaps caused by artefactual indels, the long-read-only-polished assemblies exhibited more indel variations than nt substitution changes (Fig. 2). The occurrence of these indels was significantly lower in the short- and long-read-polished data (*P*<0.0001; 95% CI=1.6535–2.1865; se=0.136). The short- and long-read-polished assemblies had an average of 0.05 indel variants (sd=0.22; median=0; max indels=1), while the long-read-only-polished assemblies had a higher average of 1.98 indel variants (sd=2.26; median=1; max indels=11).

To quantify the error rate in our dataset, we defined errors as the total number of substitutions and indels that are present in one replicate relative to another replicate in a pairwise all-against-all comparison. The genome replicates are thus deemed accurate if they have no substitutions and indels. To this end, the short- and long-read-polished genomes exhibited a mean overall error of 0.16 (sd=0.44; median=0; max=2), corresponding to an average sequence identity of 99.9999967% between any two pairs of replicates. The minimum Phred quality score was Q64.1. The long-read-only-polished genomes had a mean overall error of 2.28 (sd=2.67; median=1; max=13), corresponding to a mean overall identity of 99.9999553% with a mean Phred quality score of 63.5 (range Q54.1–Q^∞^) (Table S4). The Phred quality score was calculated as previously described [8]. We also did not detect the previously reported methylation-related errors using the MOPA tool (data not shown) [64]. One limitation of this approach is that reproducible errors that always occur at the same position between sequencing replicates were not taken into account.

These results strongly suggest substantial advancements in nanopore sequencing accuracy, even in the absence of short-read data. However, the use of short reads to polish nanopore assemblies can further improve the overall fidelity of the assembled genomes.

### High reproducibility in clustering information between replicates

Next, we sought to evaluate the impact of these artefactual substitutions and indels on the genome clustering results, as performed, e.g. in epidemiological investigations. To do so, we applied the two widely used high-resolution genotyping methods: cgSNP and cgMLST [65]. The cgSNP method aligns the sample’s genome to a reference, concatenating the identified SNPs into a genome alignment after removing sites with gaps, thereby substantially mitigating the effect of indel errors. In contrast, the cgMLST extracts the valid coding genes and compares them by blast [42] to a predefined scheme of hundreds of core genes, considering substitutions and in-frame indels, but only within the scheme core genes specific to each species [66]. The resolution of cgMLST is lower than that of cgSNP as only coding regions are considered [65,66]. We applied a hierarchical clustering approach and arbitrarily used five variants as a threshold for defining genotype clusters.

**Fig. 3.**
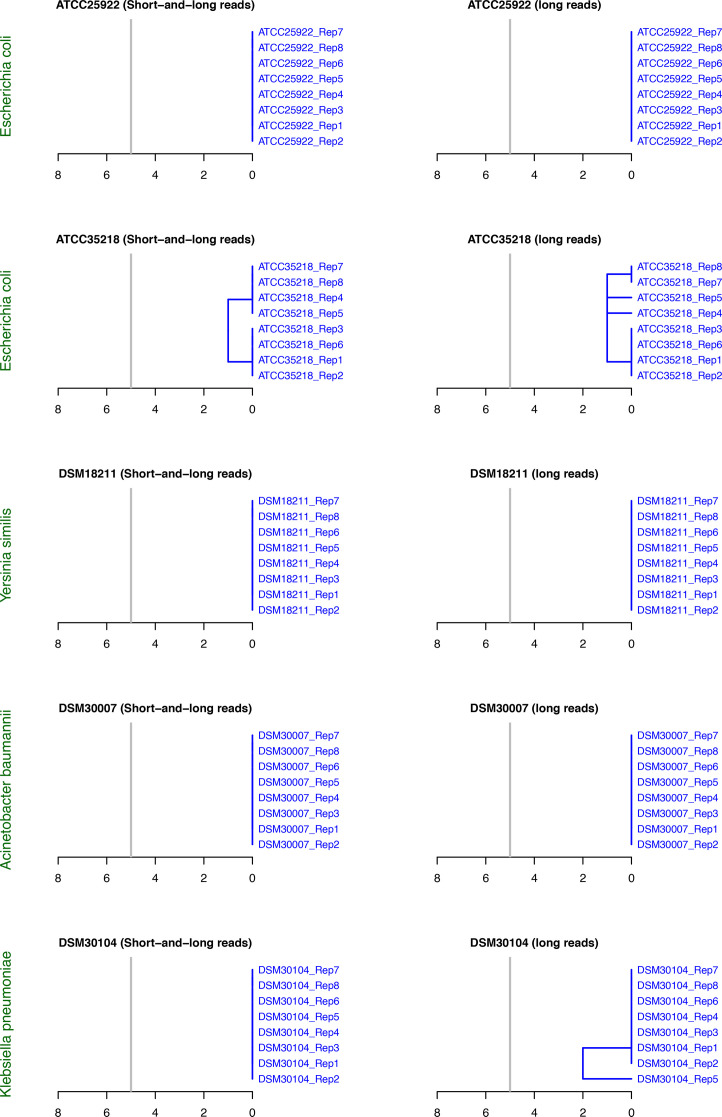
Hierarchical clustering based on cgSNP analysis. All replicates of each sample are grouped into a single cluster.

Fig. 3 shows the hierarchical clustering results based on cgSNP analysis. In the case of short- and long-read-polished assemblies, all replicates of each sample were identical, showing no SNP differences (Fig. 3). In one instance, there was one SNP between the replicates of *E. coli* ATCC 35218 (Table S5). Similarly, the long-read-only-polished assemblies showed no SNP differences between replicates of *Y. similis* DSM 18211, *E. coli* ATCC 25922 and *A. baumannii* DSM 30007 and one to two SNPs for *K. pneumoniae* DSM 30104 and *E. coli* 35218 (Table S5). The linkage SNP distance clustering showed a single robust cluster for all replicates of each sample, underscoring high accuracy and reproducibility in the genotyping results (Fig. 3).

**Fig. 4.**
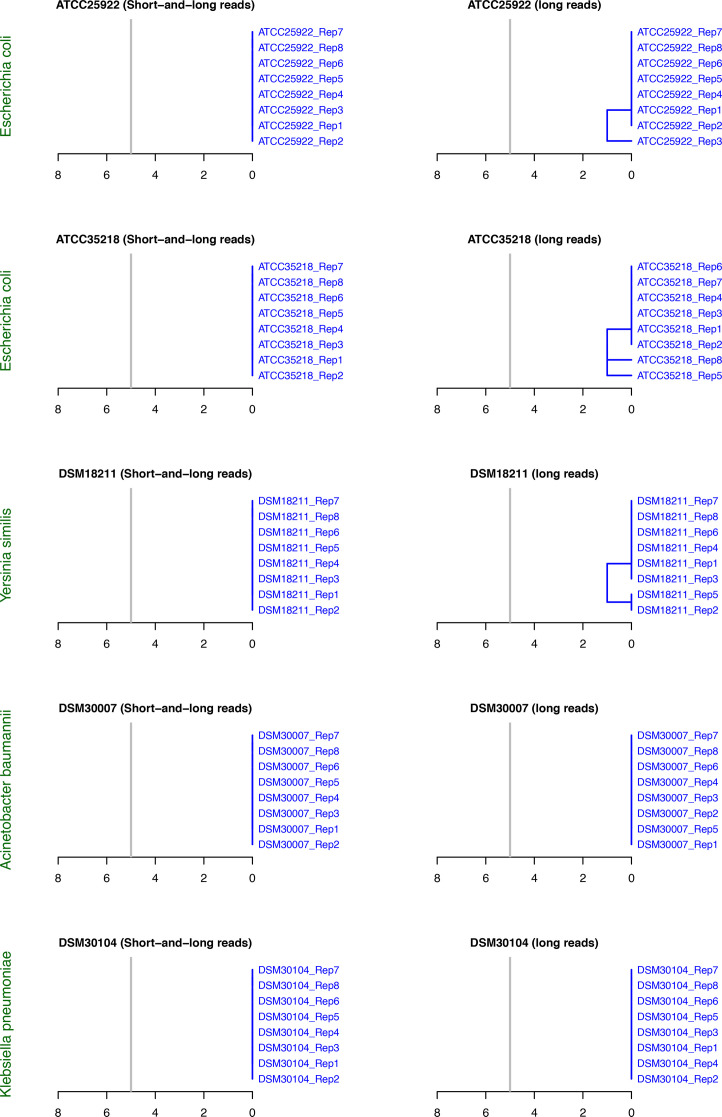
Hierarchical clustering based on cgMLST analysis. All replicates of each sample are grouped into a single cluster.

Fig. 4 shows the hierarchical clustering results from the cgMLST analysis. The cgMLST results corroborate those from the cgSNP analysis with respect to the fidelity of both approaches (Fig. 4). For the short- and long-read-polished genomes, all replicates produced identical cgMLST profiles. The cgMLST allele calling ratios were highly consistent among replicates for each sample: 96.99% for *E. coli* ATCC 25922 and *E. coli* ATCC 35218, 92.35% for *Y. similis* DSM 18211, 98.24% for * A. baumannii* DSM 30007 and 95% for *K. pneumoniae* DSM 30104. The long-read-only approach also produced similar allele calling ratios: 96.99% for *E. coli* ATCC 25922 and *E. coli* ATCC 35218, 92.35% for *Y. similis* DSM 18211, 98.20%–98.24% for *A. baumannii* DSM 30007 and 94.91%–95% for *K. pneumoniae* DSM 30104. Between sample replicates, a maximum of two pairwise allelic variants was observed, corresponding to a maximum linkage distance of one in the hierarchical clustering (Table S5, Fig. 4). The cgMLST analysis successfully grouped the replicates of each sample into a single robust cluster within the pre-set threshold of five allelic variants for clustering (Fig. 4).

In summary, the cluster analysis to identify replicates of the same strain – such as those needed in epidemiological investigations – indicates that long-read sequencing alone can provide a reliable representation of strain similarities, without short-read polishing. The clustering results demonstrated high reproducibility across all samples.

### Targeted plasmid assembly enhances reproducibility of plasmid recovery

Long-read sequencing is advantageous for reconstructing the full plasmid profile of bacterial strains. Hence, we aimed to evaluate the reproducibility in plasmid recovery between replicates. In this study, we used Flye for genome assembly and integrated an additional step with Plassembler to specifically recover plasmid sequences. Plassembler was run in its two modes: hybrid mode (combining long and short reads) and long-read-only mode. Plassembler showed improved plasmid recovery results in either mode, regardless of the sequencing kit, with both the Rapid and Ligation Barcoding kits showing comparable results. With this method, plasmid recovery was consistent and reproducible across all replicates in terms of size and number and was concordant with the NCBI reference genome sequences (Table S6). In one case, *K. pneumoniae* DSM 30104, a plasmid of size 13.2 kb (NZ_CP064369.1) was recovered in all replicates, but the reference genome was reported to carry a 24 kb plasmid (NZ_CP040992.1) (Table S6). In the long-read-only mode of Plassembler, the results were consistent for the two *E. coli* samples, but minor deviations were found for other samples. In particular, a 42.1 kb linear plasmid was missing in two replicates of *K. pneumoniae* DSM 30104, and a 7.6 kb plasmid was missing in *A. baumannii* DSM 30007. Furthermore, false contigs were detected in one replicate each of *A. baumannii* DSM 30007 and *Y. similis* DSM 18211. Overall, in the long-read-only mode of Plassembler, only 15% of all replicates (5 out of a total of 40 replicates) showed deviations from the reference genome, and 85% of the replicates were accurately aligned with the reference genome.

Despite these minor deviations, plasmid assembly with Plassembler was successful and showed superior performance compared to plasmid recovery directly from Flye assembly. The latter exhibited problems and inconsistencies in 65% of replicates across all samples (26 out of a total of 40 replicates) (Table S6). In particular, two small plasmids (1.9 and 3.2 kb) were missing in all replicates in *E. coli* ATCC 25922, two small plasmids (9.5 and 7.6 kb) were missing in six replicates of *A. baumannii* DSM 30007 and one 13.2 kb plasmid was missing in five replicates of *K. pneumoniae* DSM 30104 (Table S6). Further discrepancies include a replicate in *Y. similis* DSM 18211 with a plasmid that was 2 kb smaller than expected (size=60 kb), along with a misassembled 6.3 kb contig. In *K. pneumoniae* DSM 30104, a potential plasmid merge was observed, resulting in large fragments of 87 and 86 kb in two replicates (Table S6). In an attempt to improve the results of plasmid recovery from Flye, we used it with the flag ‘--meta’, replacing Filtlong with Rasusa [67] for down-sampling to avoid Flye failure due to excessive coverage. Although more plasmid sequences were recovered, the results between the replicates were largely inconsistent with frequent plasmid mis-assembly problems (Table S6).

Our findings agree with previous studies showing that long-read assemblers, such as Flye and Raven [68], often fail to accurately reconstruct small plasmids [19,69]. Long-read assemblers can also erroneously merge plasmid sequences or generate multiple copies of a single plasmid, resulting in spurious and inconsistent plasmid recovery, a problem observed in our Flye assemblies. Despite these issues, Flye has been reported in a previous study as one of the better long-read assemblers for plasmid recovery compared to others such as miniasm [70], Raven [68] and Canu [71]. It is also important to note that the Ligation sequencing kit has been reported to miss small plasmids due to adapter binding issues, whereas the Rapid sequencing kits are generally more effective [72]. However, in our dataset, the outcome of plasmid recovery was more dependent on the assembly workflow than the sequencing approach. In summary, the targeted plasmid assembly implemented in Plassembler [30] improved the overall reproducibility of plasmid recovery, including small plasmids.

### Robust identification of genetic markers using nanopore-only assemblies without short-read polishing

In the next steps, we aimed to evaluate the reproducibility of various genomic analyses across replicates. The results are summarized in Table S7.

#### Antimicrobial resistance

The results from detecting genetic determinants of antimicrobial resistance showed high reproducibility, regardless of the short reads (Table S7). Both the long-read-only-polished and the short- and long-read-polished assemblies resulted in consistent identification of genetic markers of antimicrobial resistance. For *A. baumannii* DSM 30007, ABRicate consistently identified 8 genes with ARG-ANNOT, 22 with CARD, 26 with MEGARes, 4 with NCBI and 3 with ResFinder in all replicates. AMRFinderPlus detected four resistance genes, while the RGI_CARD detected 24 resistance markers (23 with the protein homologue model and 1 with the protein variant model). Similarly, for *K. pneumoniae* DSM 30104, ABRicate reported 6 genes with ARG-ANNOT, 27 with CARD, 35 with MEGARes, 4 with NCBI and 4 with ResFinder, with AMRFinderPlus identifying four resistance genes. The RGI_CARD identified 34 resistance markers, including 29 with the protein homologue model, 3 with the protein variant model and 2 with the protein overexpression model. In *E. coli* ATCC 25922, ABRicate detected 3 genes with ARG-ANNOT, 43 with CARD, 53 with MEGARes, 1 with NCBI and none with ResFinder. AMRFinderPlus identified two point mutations conferring resistance. The RGI_CARD identified 55 resistance markers (47 with the protein homologue model, 3 with the protein variant model and 5 with the protein overexpression model). For *E. coli* ATCC 35218, ABRicate identified 8 genes with ARG-ANNOT, 48 with CARD, 57 with MEGARes, 6 with NCBI and five with ResFinder, while AMRFinderPlus detected six resistance genes and two point mutations conferring resistance. The RGI_CARD identified 60 resistance markers (52 with the protein homologue model, 3 with the protein variant model and 5 with the protein overexpression model). In *Y. similis* DSM 18211, no resistance genes were detected by either ABRicate (with ARG-ANNOT, NCBI or ResFinder) or AMRFinderPlus, but ABRicate identified three genes with each of CARD and MEGARes databases. RGI_CARD detected ten resistance markers (eight with the protein homologue model and two the with protein variant model). These results were consistent in all replicates.

#### Virulence markers

Virulence factor detection also showed high consistency across all replicates (Table S7). ABRicate with the VFDB identified 109 virulence genes in *A. baumannii* DSM 30007, 94 in *K. pneumoniae* DSM 30104, 129 in *E. coli* ATCC 25922, 132 in * E. coli* ATCC 35218 and 20 in *Y. similis* DSM 18211. These numbers were consistently identified in all replicates of each sample. Additionally, for the *E. coli* strains ATCC 25922 and ATCC 35218, a serovar prediction was performed using the ECTyper tool. The strains were assigned with high confidence to serotypes O6:H1 and O6:H31, respectively, in all replicates. For the *K. pneumoniae* sample DSM 30104, the Kleborate tool was applied. Kleborate detected genes associated with hypervirulence, including yersiniabactin, colibactin and aerobactin. Kleborate assigned the strains a virulence score of 2 (based on the presence of genes encoding the known virulence factors yersiniabactin, colibactin and aerobactin) and a resistance score of 0. No differences were observed between replicates, except for the hypermucoidy-associated rmpADC locus. While the rmp2A lineage was consistently identified across all samples, the rmST calling was affected by indel errors. Characterization of the capsular locus and LPS O antigen, performed with Kaptive, also revealed no differences between replicates (Table S7).

#### Classical MLST

MLST analysis consistently classified all replicates of each strain into the respective MLST type (Table S7). Replicates of DSM 30007 were classified with the abaumannii_2 scheme as ST52, DSM 30104 as ST3 with the *Klebsiella* scheme and DSM 18211 as ST92 with the ypseudotuberculosis_achtman_3 scheme. The *E. coli* strains ATCC 25922 and ATCC 35218 were classified with the ecoli_achtman_4 scheme as ST73 and ST127, respectively. The MLST results were consistent across all replicates, with and without short-read polishing.

#### Taxonomic classification

Kraken and FastANI analyses accurately classified all samples to their respective species (Table S7). Kraken assignment percentages to taxonomic clades were consistent and absolute (100%) for the samples ATCC 25922, ATCC 35218 and DSM 30104 for the species *E. coli* and *K. pneumoniae*, respectively. A slight variation in this percentage was found in one replicate of the samples * Y. similis* DSM 18211 and *A. baumannii* DSM 30007 (66% and 75%), due to the spurious contig sequences (Table S6). ANI analysis confirmed ANI matches above 99% to the reference genome in all replicates (Table S7).

#### Genome completeness and contamination

The completeness and contamination score as reported by CheckM showed no variation across replicates (Table S7). CheckM reported 99.97% completeness and 0.12% contamination, with no strain heterogeneity for *E. coli* ATCC25922. In *E. coli* ATCC35218, CheckM indicated 99.97% completeness and 0.07% contamination, with no strain heterogeneity. DSM 18211 (*Y. similis*) was reported by CheckM with 99.92% completeness and 0.87% contamination, with no strain heterogeneity noted. For *A. baumannii* DSM 30007, CheckM indicated 99.63% completeness and 0% contamination. For *K. pneumoniae* DSM 30104, CheckM results showed 100% completeness and 0.97% contamination with 16.67% strain heterogeneity.

#### Pan-genome clustering

Pan-genome clustering, which identifies accessory genomes and creates distribution-based trees, is a common approach for describing relationships between strains, particularly useful for informative outbreak analyses [73,74]. This process typically involves creating a pan-genome profile by clustering similar gene families. When analysing replicates of a single genome, one would expect no accessory genomes to be identified. To investigate this, we used Panaroo for gene clustering. Panaroo predicted no accessory genomes in the replicates of short- and long-read-polished assemblies. Similarly, Panaroo predicted no accessory genes in the long-read-only assemblies of *E. coli* ATCC 25922, *E. coli* ATCC 35218 and *Y. similis* DSM 18211 but reported 12 and 52 accessory genes for *A. baumannii* DSM 30007 and *K. pneumoniae* DSM 30104, respectively. These incorrect predictions of accessory genomes are primarily due to the inconsistent plasmid recovery results, notably the missing plasmids.

Taken together, the results demonstrate that both the short- and long-read and long-read-only approaches yield highly consistent results across multiple replicates for resistance and virulence marker identification, classic MLST, taxonomic classification and genome completeness assessment. The pan-genome estimation and inference of the accessory genome tree were accurate with the long-read-only data but influenced by the plasmid recovery results.

## Conclusion

The reproducibility of next-generation sequencing is essential to ensure data reliability and support accurate conclusions. At the forefront of sequencing technologies in bacterial studies are Illumina and nanopore, each with its own strengths. Illumina uses clustering, sequencing by synthesis and large-scale bridge amplification and is known for its accuracy. Nanopore utilizes single-molecule technology and enables sequencing of long reads. However, the accuracy of nanopore sequencing is a matter of concern.

Our results demonstrate that nanopore-only assemblies achieve remarkable accuracy, with an average error rate of 2.2 variants (indels and substitution) per genome (sd 2.6). This accuracy was independent of the application of Ligation or Rapid Barcoding kits and was evident on Flongle and MinION flow cells. Nanopore-only assemblies showed high reproducibility in various genomic analyses, such as the identification of genetic markers for antimicrobial resistance and virulence, classical MLST, taxonomic classification and assessing genome completeness and contamination. Moreover, cluster analyses, which are crucial for outbreak investigations, showed highly consistent results for nanopore-only assemblies. The cgSNP and cgMLST analyses showed minimal deviations between replicates, with pairwise differences limited to a maximum of two SNPs for cgSNP analyses and two allelic differences for cgMLST. This accuracy allows for a rigorous delineation of genetic relationships between isolates, e.g. in epidemiological studies. The improved accuracy of nanopore assemblies was also very recently reported by others [11]. Yet, the *de novo* reconstruction of plasmids was challenging, as long-read assemblers such as Flye often face difficulty with recovering small plasmids and may misassemble plasmid sequences [69].

Limitations of our study include the restriction of our analysis to a few clinically important species (*n*=4), with a single DNA preparation from a reference strain analysed per species. While this approach was intentional and important to assess the reproducibility of sequencing and mitigate the effects of sub-culturing, it may limit the generalizability of our findings. Furthermore, we used a reference-free approach to assess genome accuracy by comparing all sequencing replicates in pairs. This approach discounts errors that occur at the same position in all replicates. Finally, although we carefully optimized our assembly workflow to maximize accuracy, we note that continued development of nanopore data processing software is likely to yield tools that can further improve overall performance in the future.

## supplementary material

10.1099/mgen.0.001372 Uncited Fig. S1.

10.1099/mgen.0.001372 Uncited Supplementary Material 1.

## Abbreviations

ANI: average nucleotide identity
cgMLST: core genome MLST
cgSNP: core genome SNPs
RGI: resistance gene identifier
VFDB: virulence factor database
