# Supplementary material for: High intra-laboratory reproducibility of nanopore sequencing in bacterial species underscores advances in its accuracy
Source: Microb Genom. 2025 Mar 21;11(3):001372. doi: 10.1099/mgen.0.001372 (PMC11927881; doi:10.1099/mgen.0.001372)
Supplement: Uncited Fig. S1. [file mgen-11-01372-s001.pdf]

Supplementary Figure 1: (A) Overview of the workflow for the assembly of Oxford Nanopore sequencing data of bacterial genomes. (B) Directed acyclic diagram of the steps implemented in the study for the short- and long-read approach (B) and the long-read approach (C).

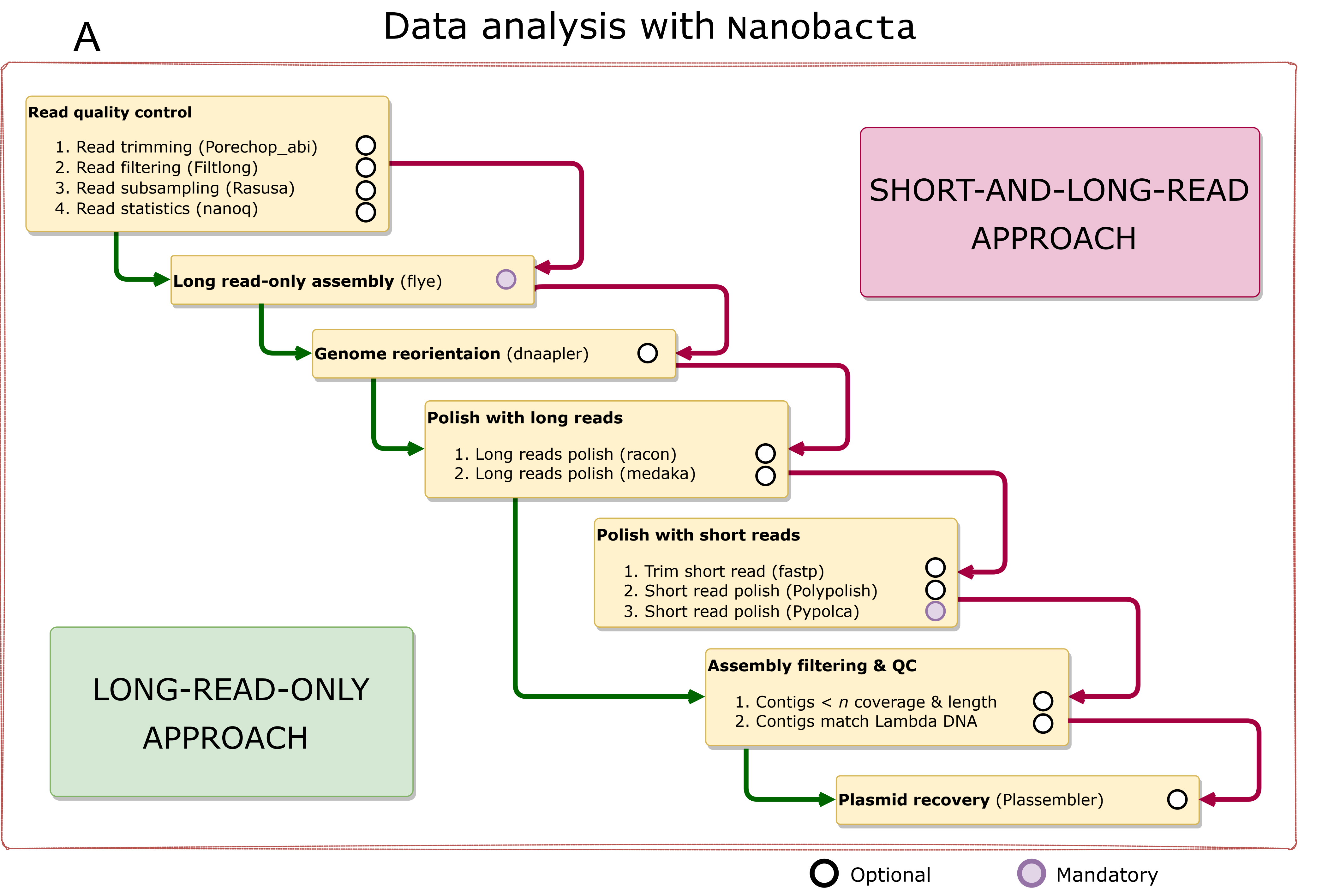
A
Data analysis with Nanobacta
Read quality control
1. Read trimming (Porechop_abi)
2. Read filtering (Filtlong)
3. Read subsampling (Rasusa)
4. Read statistics (nanoq)
SHORT-AND-LONG-READ APPROACH
Long read-only assembly (flye)
Genome reorientaion (dnaapler)
Polish with long reads
1. Long reads polish (racon)
2. Long reads polish (medaka)
Polish with short reads
1. Trim short read (fastp)
2. Short read polish (Polypolish)
3. Short read polish (Pypolca)
LONG-READ-ONLY APPROACH
Assembly filtering & QC
1. Contigs < n coverage & length
2. Contigs match Lambda DNA
Plasmid recovery (Plassembler)
Optional
Mandatory

B

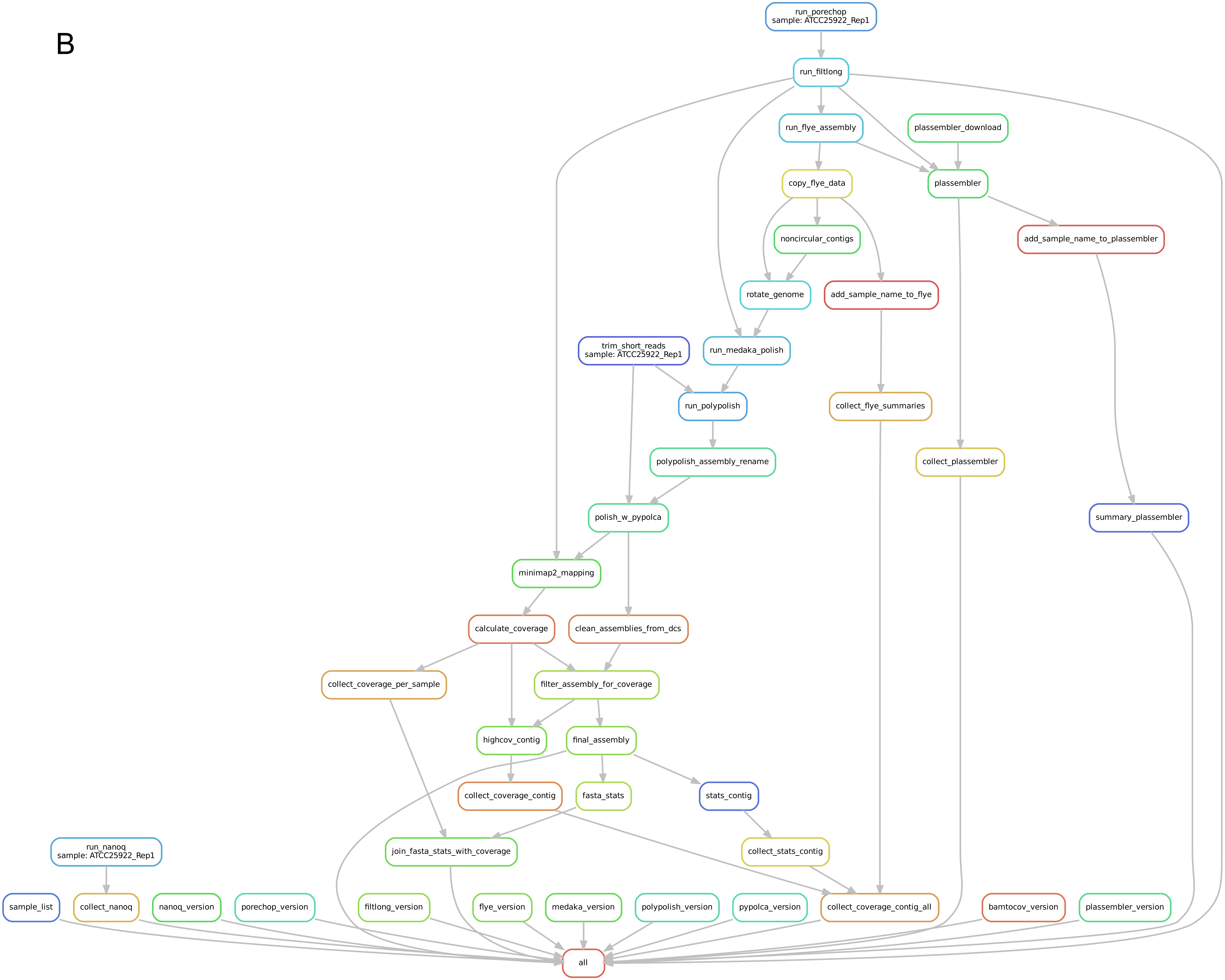
run_porechop
sample: ATCC25922_Rep1
run_filtlong
run_flye_assembly
plassembler_download
copy_flye_data
plassembler
noncircular_contigs
add_sample_name_to_plassembler
rotate_genome
add_sample_name_to_flye
trim_short_reads
sample: ATCC25922_Rep1
run_medaka_polish
run_polypolish
collect_flye_summaries
polypolish_assembly_rename
collect_plassembler
polish_w_pypolca
summary_plassembler
minimap2_mapping
calculate_coverage
clean_assemblies_from_dcs
collect_coverage_per_sample
filter_assembly_for_coverage
highcov_contig
final_assembly
collect_coverage_contig
fasta_stats
stats_contig
run_nanoq
sample: ATCC25922_Rep1
join_fasta_stats_with_coverage
collect_stats_contig
sample_list
collect_nanoq
nanoq_version
porechop_version
filtlong_version
flye_version
medaka_version
polypolish_version
pypolca_version
collect_coverage_contig_all
bamtocov_version
plassembler_version
all

C

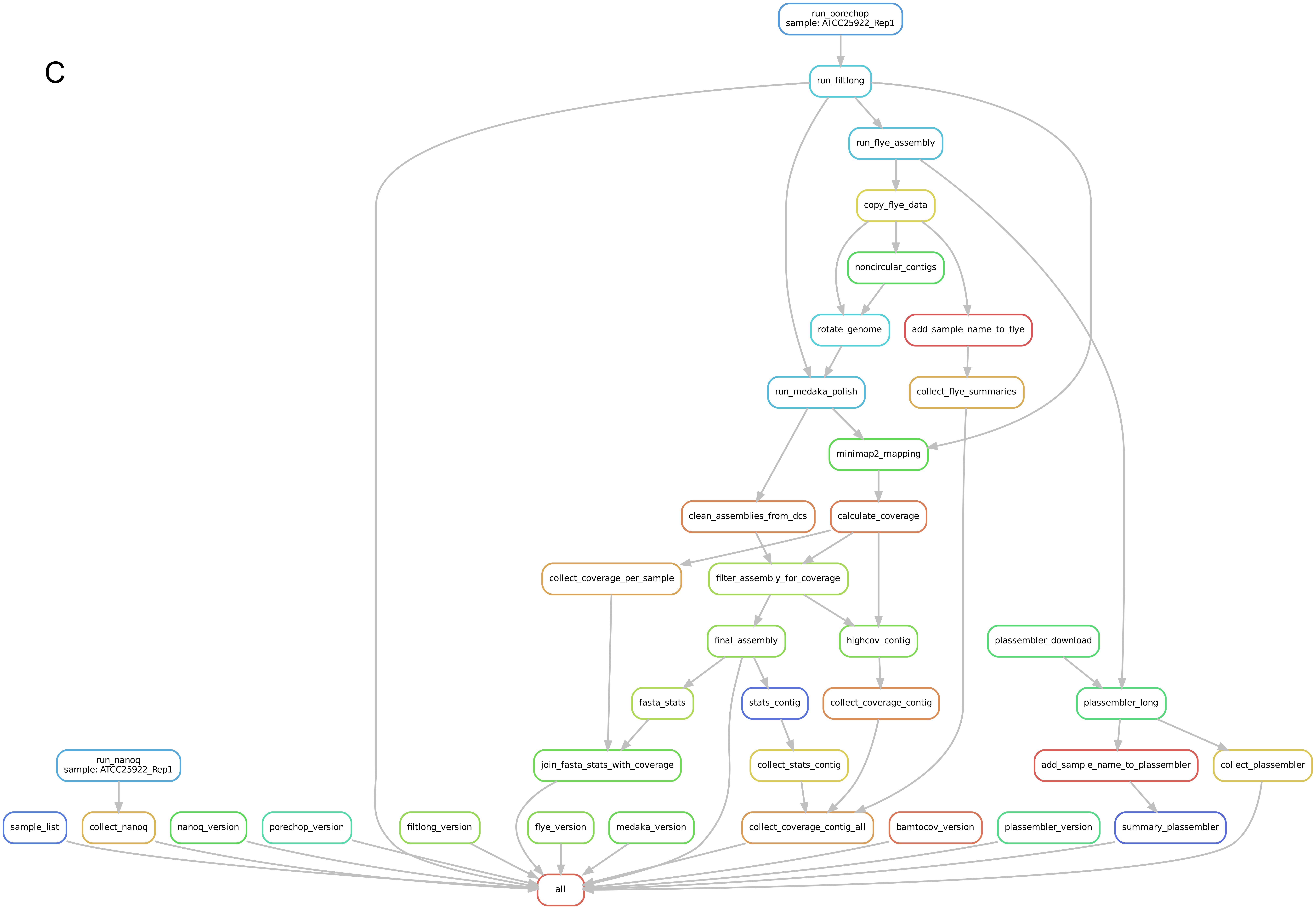

run_porechop
sample: ATCC25922_Rep1
run_filtlong
run_flye_assembly
copy_flye_data
noncircular_contigs
rotate_genome
add_sample_name_to_flye
run_medaka_polish
collect_flye_summaries
minimap2_mapping
clean_assemblies_from_dcs
calculate_coverage
collect_coverage_per_sample
filter_assembly_for_coverage
final_assembly
highcov_contig
plassembler_download
fasta_stats
stats_contig
collect_coverage_contig
plassembler_long
run_nanoq
sample: ATCC25922_Rep1
join_fasta_stats_with_coverage
collect_stats_contig
add_sample_name_to_plassembler
collect_plassembler
sample_list
collect_nanoq
nanoq_version
porechop_version
filtlong_version
flye_version
medaka_version
collect_coverage_contig_all
bamtocov_version
plassembler_version
summary_plassembler
all
